# Characterization of cardiac autonomic dysfunction in acute Schizophrenia: a cluster analysis of heart rate variability parameters

**DOI:** 10.1038/s41537-025-00589-y

**Published:** 2025-03-08

**Authors:** Alexander Refisch, Andy Schumann, Yubraj Gupta, Steffen Schulz, Andreas Voss, Berend Malchow, Karl-Jürgen Bär

**Affiliations:** 1 Department of Psychiatry and Psychotherapy, Jena University Hospital, Jena, Germany; 2 Circuits Underlying Mental Health (C-I-R-C), Jena-Magdeburg-Halle, 07743 Jena, Germany; 3 Lab for Autonomic Neuroscience, Imaging and Cognition (LANIC), Department of Psychosomatic Medicine and Psychotherapy, Jena University Hospital, Jena, Germany; 4 Charité Competence Center for Traditional and Integrative Medicine (CCCTIM), Charité—Universitätsmedizin Berlin, Corporate Member of Freie Universität Berlin, Humboldt-Universität zu Berlin and Berlin Institute of Health, Berlin, Germany; 5 Institute of Biomedical Engineering and Informatics (BMTI), Technische Universität Ilmenau, Ilmenau, Germany; 6 Department of Psychiatry and Psychotherapy, University Hospital Göttingen, Göttingen, Germany

**Keywords:** Biomarkers, Schizophrenia

## Abstract

Underlying biological mechanisms leading to the dramatically increased cardiac mortality in patients with schizophrenia (SCZ) are largely unknown. Cardiac autonomic dysfunction (CADF), which has been extensively described in patients with SCZ, represents an important physiological link to cardiovascular disease (CVD). This study investigated the prevalence of CADF in patients with SCZ using HRV across multiple domains (time and frequency, nonlinear dynamics, complexity measures, symbolic dynamics, and segmented Poincaré plot analysis). HRV-based clustering classified 119 SCZ patients as having or not having CADF based on deviations from 119 age- and sex-matched healthy controls. Our findings showed that approximately half of the patients had normal cardiac autonomic function, while the other half had significant abnormalities. The severity of CADF correlated with age, body mass indes (BMI), disease duration, and symptom severity. About half of SCZ patients have significant CADF, which increases their risk for cardiac events. These findings highlight the potential of HRV-based biomarkers in improving CVD risk prediction and stratification in SCZ. Future research should explore integrating HRV analysis with other biomarkers to enhance early detection and intervention strategies.

## Introduction

Schizophrenia (SCZ) is associated with a significant reduction in life expectancy of 15–20 years compared to the general population^1–3^, with cardiovascular disease (CVD) being a leading cause of death in this population^4–7^.

A key contributor to this elevated cardiovascular risk is cardiac autonomic dysfunction (CADF), characterized by decreased vagal and increased sympathetic modulation, which is an independent risk factor for premature mortality and life-threatening arrhythmias in individuals with or without structural heart disease^8–10^. This dysregulated autonomic balance, consistently observed in SCZ patients^11^, plays a critical role in the development and progression of major CVD, such as myocardial infarction, heart failure, and sudden cardiac death^12–15^. Understanding and addressing CADF in SCZ may therefore offer valuable insights for cardiovascular risk stratification and prevention of adverse outcomes in SCZ patients.

Notably, CADF in patients with SCZ reflects not merely transient stress-induced arousal, but persistent underlying alterations in autonomic regulation^16^. On the one hand, CADF in first-degree relatives suggests a genetic predisposition^17^, which is further supported by SCZ-associated loci that regulate cardiac function^18,19^. On the other hand, while general contributors such as smoking^20^, BMI^21^, and age^22–24^ are well-documented, more specific SCZ-associated factors such as chronic stress, systemic inflammation, and metabolic dysregulation may further exacerbate CADF^25^. Beyond these general factors, antipsychotic medication use and symptom severity must also be considered, as they may uniquely interact with SCZ’s underlying pathophysiology. Understanding the multifactorial nature of CADF, including both general and SCZ-associated factors, and its interactions with genetic influences is essential to develop accurate assessments to quantify cardiac autonomic risk in this vulnerable population.

A key marker of CADF is heart rate variability (HRV), which reflects the dynamic interplay between sympathetic and parasympathetic modulation and serves as a noninvasive assessment of autonomic regulation, with higher variability generally indicating better cardiovascular adaptability and overall health^26^.

Classical HRV analysis includes time-domain measures that quantify the variability of RR intervals over time (e.g., SDNN for overall variability and RMSSD for parasympathetic activity) and frequency-domain measures that assess the distribution of power across frequency bands (e.g., LF and HF) to evaluate autonomic balance^27^. However, nonlinear HRV measures provide deeper insights into the complexity and irregularity of heart rate dynamics^28,29^. These include entropy measures, which quantify randomness^30,31^, symbolic dynamics, which analyzes variability and complexity in RR interval patterns^32,33^; Poincaré plot analysis, which evaluates short- and long-term variability^34,35^; and compression entropy, which measures signal complexity^32,36^. Integrating classical HRV measures with nonlinear dynamics enhances sensitivity in detecting subtle autonomic dysfunction^37–39^. While classical parameters provide robust markers of overall autonomic activity, nonlinear measures uncover hidden patterns, providing a more comprehensive view of cardiac regulation^16,29^.

Despite extensive research on CADF in SCZ, previous studies have been limited by small sample sizes or confounded by the effects of antipsychotic medication^25^. To address these limitations, the current study examines a robust cohort of 119 unmedicated SCZ patients using a comprehensive set of HRV parameters to evaluate the prevalence of CADF compared to healthy controls.

We hypothesize that CADF is highly prevalent in unmedicated SCZ patients and that its severity correlates with advanced age, increased BMI, severity of SCZ positive and negative symptoms, and longer disease duration. This study aims to deepen our understanding of the autonomic dysfunction underlying SCZ and identify risk characteristics for CADF, ultimately contributing to improved cardiovascular risk assessment and management in this population.

## Methods

### Participants

A total of 119 patients with SCZ from the Department of Psychiatry and Psychotherapy at the University Hospital Jena participated in this single-center investigation between January 2010 and December 2017 (see Supplementary Table S1). Inclusion criteria required a diagnosis of SCZ according to the Diagnostic and Statistical Manual of Mental Disorders, 4th revision (DSM-IV) criteria, an age between 18 and 45 years, and a minimum of antipsychotic-free period of at least 8 weeks before admission to the hospital. Moreover, Patients had to be legally competent. The Brief Structured Diagnostic Interview for Major Psychiatric Disorders (SCID)^40^ was administered by a staff psychiatrist to identify relevant psychiatric comorbidities and to determine disease duration in patients with SCZ. The diagnosis of SCZ was independently confirmed by another psychiatrist. Exclusion criteria included psychiatric comorbidities (e.g., current substance abuse or dependence, assessed by urine drug screen), clinically relevant medical conditions (e.g., cardiovascular, peripheral nervous system, or endocrine disorders, assessed by clinical interview and review of medical records), involuntary hospitalization, or pregnancy. Symptom severity was assessed using the *Scale for the Assessment of Negative Symptoms (SANS)*^41^, and the *Scale for the Assessment of Positive Symptoms (SAPS)*^42^.

Data were collected cross-sectionally within one week after hospital admission. Inclusion in the study did not affect the clinical course or treatment decisions of the participants. Routine psychotherapeutic crisis interventions, such as supportive conversations may have occurred as part of standard care, but no specific stress-reduction therapies, such as biofeedback, relaxation exercises, or guided breathing techniques, were administered.

In addition, we recruited 119 healthy controls (HC) matched for age, gender, and BMI, from the hospital staff, medical students and the local community. The SCID was performed to exclude any current or past psychiatric disorders, and somatic diseases were ruled out through clinical interview. Participants taking medications affecting the cardiovascular or autonomic nervous system (e.g., beta-blockers, antiarrhythmics, antipsychotics, antidepressants) were excluded.

All participants underwent a comprehensive clinical examination, ECG assessment and a routine laboratory testing.

The study was approved by the local ethics committee and conducted in accordance with the Declaration of Helsinki. All participants provided written informed consent, and patients were assured that declining participation at any time would not affect future treatment.

### Analysis of heart rate variability parameters

#### ECG recording and Data preparation

A high-resolution 3-channel electrocardiogram (1000 Hz) was obtained from all participants for 30 min using a Task Force® Monitor (CNSystems Medizintechnik AG, Graz, Austria). The recording was conducted in a quiet room with stable air temperature maintained at 22 ± 2 °C. Participants were asked to breathe evenly, move as little as possible, to not talk and avoid falling asleep. Furthermore, patients with SCZ as well as HC were asked to avoid physical strain, eating large meals and the use of nicotine or caffeine for 2 h prior to the ECG recording.

The ECG was acquired by arranging three electrodes on the chest according to an adjusted Einthoven triangle. ECG signals were band-pass filtered between 0.05 and 35 Hz. Automatically detected R-waves were manually reviewed for ectopic beats and artifacts. Missed heartbeats were identified, and artifacts were removed based on the morphology of QRS complexes in the ECG signals. The resulting heartbeat interval time series was further screened for outliers using adaptive filtering^43^. Detected abnormal intervals were corrected by replacing them with randomized values within a range defined by the mean and standard deviation of the neighboring heartbeat intervals. This approach minimizes systematic influences on specific HRV parameters, which have been shown to occur when linear or spline interpolation was used after artifacts removal^44,45^.

Due to the high scanning frequency and thus temporal resolution of the ECG recordings the obtained measures regarding heart beat intervals allow a reliable calculation of parameters of HRV^46^. The calculation of HRV parameters was based on the time series of normal-to-normal (NN) intervals derived from ECG signals, processed as described above.

To comprehensively capture abnormalities and changes in heart rate patterns over time, we integrated parameters from various domains into our model, each providing distinct insights into the dynamics of heart rate time series. The following is a brief overview of each domain. For a detailed description of domains, see ***Supplementary material*** information.

### Time domain analysis

HRV is assessed by measuring the intervals between successive heartbeats, known as NN intervals (normal-to-normal intervals) or RR intervals (time between two R waves on an ECG). Traditional time and frequency domain parameters for describing HRV are well defined and widely accepted^27^. The time domain approach provides valuable insight into autonomic cardiac regulation by the sympathetic and parasympathetic nervous systems using parameters such as standard deviation of NN intervals (SDNN), reflecting the total variability in heart rate, and root mean square of successive differences (RMSSD), specifically measuring short-term variability driven by vagal influences^47^. While mean heart rate (HR) is not a direct HRV parameter, it is derived from time-domain data. Given its fundamental importance, HR was included in our analysis.

### Frequency domain analysis

HRV signals are decomposed into frequency bands using spectral analysis methods such as Fast Fourier Transformation^48^. High frequency (HF: 0.15–0.4 Hz) components indicate vagal modulation, while low frequency (LF: 0.04–0.15 Hz) components reflect both sympathetic and vagal influences, including baroreflex activity. The LF/HF ratio represents the balance of autonomic inputs. These parameters provide a detailed understanding of cardiac autonomic modulation.

### Multiscale entropy (MSE)

MSE, introduced by Costa and colleagues, assesses HRV complexity by measuring entropy across multiple time scales^30^. The time series is coarse-grained by averaging successive data points over increasing windows. The sample entropy (SampEn) is calculated for each time scale to quantify regularity and complexity. Higher SampEn values indicate more complex and less predictable HR patterns, reflecting robust physiological regulation.

### Detrended fluctuation analysis (DFA)

Trend-adjusted fluctuation analysis is a widely used method for the quantification of correlations in nonstationary HRV time series^49^. RR intervals are integrated, segmented, and detrended by removing local linear trends. The fluctuation function F(n) is calculated for each segment length n, and the scaling exponent *alpha*, derived from the slope of a log-log plot of F(n) versus n, represents correlation properties. Short-term scaling *alpha1* (4–16 intervals) and long-term scaling *alpha2* provide insight into autonomic dynamics and system adaptability.

### Compression entropy (Hc)

Hc quantifies the compressibility and complexity of RR intervals using the LZ77 algorithm^50^. It is calculated as the ratio of the compressed length to the original length of the time series. A higher Hc indicates greater complexity, reflecting a diverse and adaptive heart rate regulation.

### Symbolic dynamics

The symbolic dynamics method analyzes the temporal dynamics of HRV by comparing each heartbeat to the previous one^51^. RR intervals are converted into symbols representing changes (e.g., increases, decreases, or stability), and word sequences derived from these symbols provide insight into the dynamics of the system. Entropic measures, such as Rényi entropy, and specific word distributions quantify nonlinear variability and complexity. Reduced symbol sets, as proposed by Voss and colleagues, allow the identification of high and low variability states, enhancing the analysis of regulatory patterns^52^.

### Poincaré plot analysis

Trend-adjusted fluctuation analysis is widely used to quantify correlations in nonstationary series^49^. This nonlinear method visualizes the relationship between successive RR intervals by plotting each interval against the next. The resulting scatterplot provides qualitative and quantitative measures of variability. An ellipse fitted to the plot quantifies short-term variability (SD1) and long-term variability (SD2). Advanced methods include rotation and segmentation of the plot to analyze specific trends and patterns in HRV dynamics, providing deeper insight into autonomic regulation.

### Clustering CADF phenotypes by using HRV parameters

**Fig. 1:**
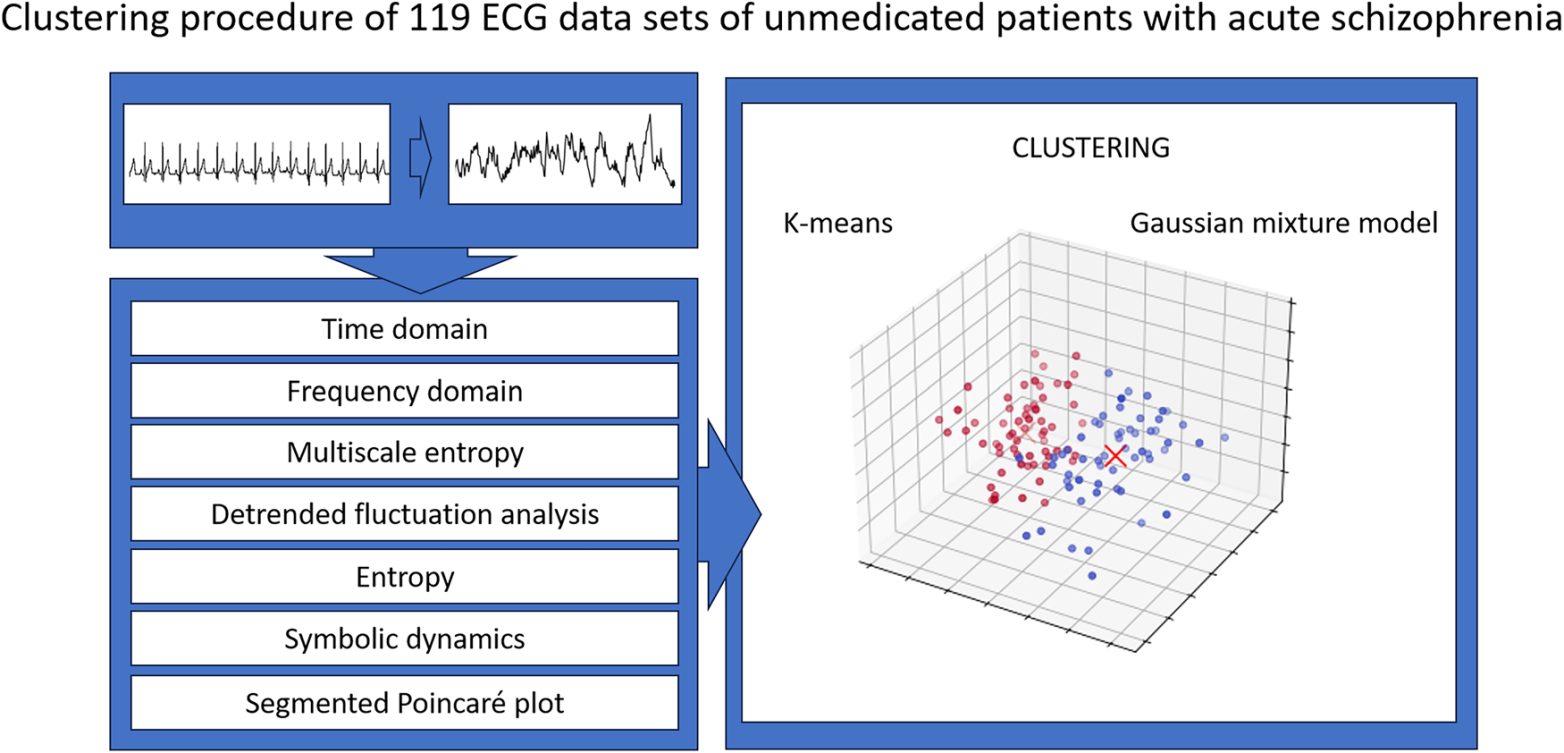
Schematic representation of the clustering procedure. Heartbeats were extracted from ECG recordings for all participants, and HRV parameters from multiple domains (e.g., time, frequency, and nonlinear dynamics) were derived from the interbeat interval series. These features were then used for clustering, with k-means and Gaussian mixture models applied to classify patient samples into distinct subgroups.

To quantify the extent of CADF in unmedicated patients with SCZ, we applied an unsupervised machine learning clustering algorithm to HRV parameters from the previously described domains (Fig. 1). Supplementary Table S2 summarizes all 97 HRV parameters analyzed in this study.

First, we scanned all HRV parameters for outliers using a median absolute deviation threshold of 10%. An imputation strategy using mean values was employed to replace missing or zero values. This step ensured a complete dataset, which is crucial for subsequent analysis. Next, the data was standardized using robust range scaling to normalize the feature set.

The *k-means* and Gaussian Mixture Model (GMM) algorithms were used to partition data sets according to HRV parameters into a defined number of clusters^53^. K-means is the most-used clustering technique for its simplicity and efficiency. Gaussian processes provided a more flexible, probabilistic approach, which was useful in capturing potential non-linear relationships and uncertainties in the data. In k-means, clusters of data points were defined by cluster centroids that have small distances to other points of this cluster but large distances to centroids of other clusters. In an iterative process, data points were assigned to their nearest cluster centroid and optimal cluster centroids were re-calculated. The process continues, reducing the sum of distances after each reassignment to minimize the sum of point-to-centroid distances.

GMM assumes that the data points within each cluster are generated from a mixture of several Gaussian distributions with unknown parameters. Unlike k-means, where each data point is assigned exclusively to one cluster, GMM allows for soft clustering. This means that each data point is assigned a probability distribution over all clusters, reflecting the uncertainty about its cluster assignment. This flexibility can be advantageous when dealing with data that doesn’t have clearly defined boundaries between clusters.

We determined the optimal number of clusters by systematically evaluating several established clustering performance indices. The Silhouette Score was used to assess the compactness and separation of clusters, with higher scores indicating better-defined clusters and the peak score corresponding to the most appropriate number of clusters. The Calinski-Harabasz Index evaluated the ratio of between-cluster dispersion to within-cluster dispersion, with higher values reflecting more distinct clustering structures. The Davies-Bouldin Index measured the average similarity between each cluster and its most similar counterpart, where lower values indicated better separation and compactness. To identify the optimal clustering solution, we evaluated these indices across a range of cluster counts (k) from 2 to 14 separated by k-means. The final number of clusters was determined by selecting the k value at which the Silhouette Score peaked, further validated by maximizing the Calinski-Harabasz Index and minimizing the Davies-Bouldin Index.

The hyperparameters for each clustering method, such as the initialization method, the number of initializations for K-means, and the initialization parameters and covariance type for GMM, were meticulously optimized, ensuring the robustness of our methodology. All algorithms used here are part of the scikit-learn (version 1.2.2) package implemented in python (version 3.11.3)^54^.

### Statistical analysis

Statistical analyses were performed using SPSS for Windows (version 26.0). Multivariate analysis of variance (MANOVA) was performed to assess differences in HRV parameters between diagnostic groups (SCZ vs. HC). To evaluate HRV differences between patient clusters (CADF vs. no CADF), age and sex were included as covariates in the MANOVA.

To determine which HRV parameters contributed to CADF cluster classification, a bivariate Spearman correlation analysis was performed correlating each HRV feature with CADF group assignment (group = 1 for CADF, group = 0 for no CADF).

In addition, sociodemographic factors, psychometric scales, and clinical characteristics, including age, BMI, physical activity, smoking status, disease duration, and symptom severity (measured by SANS and SAPS) were compared between CADF clusters. Spearman correlation analysis was also performed to examine associations between sociodemographic variables, psychometric measures, and cluster assignment, with a focus on age, body mass index (BMI), disease duration, and symptom severity.

## Results

### Study sample and group comparisons of HRV parameters

Our sample consisted of 119 patients (52% female, mean age 32.8 years) and 119 healthy controls (45% female, mean age 31.5 years; see Supplementary Table S1 for details).

The MANOVA showed a significant effect of diagnosis (F = 2.306, *p* < 0.001) on HRV parameters.

### Prevalence of CADF in unmedicated patients with schizophrenia based on HRV clustering

**Fig. 2:**
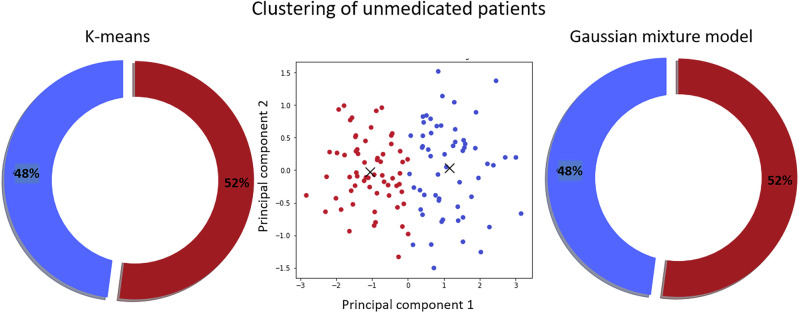
Pie chart illustrating the classification of 119 unmedicated patients with schizophrenia based on a comprehensive analysis of heart rate variability parameters (percentages shown). Two clustering techniques, k-means (left) and Gaussian mixture model (right), produced identical patient group separations. The middle panel visualizes the clustering using the first two principal components.

After the automatic clustering of our data set, the cohort of 119 unmedicated patients with SCZ was divided into two clusters as indicated by different methods to derive the optimal number of clusters. K-means and Gaussian mixture models revealed the identical separation into two clusters containing 52.1% (CADF) and 47.9% (no CADF) of patients with SCZ (see Fig. 2).

### Differences in HRV parameters between clusters

**Fig. 3:**
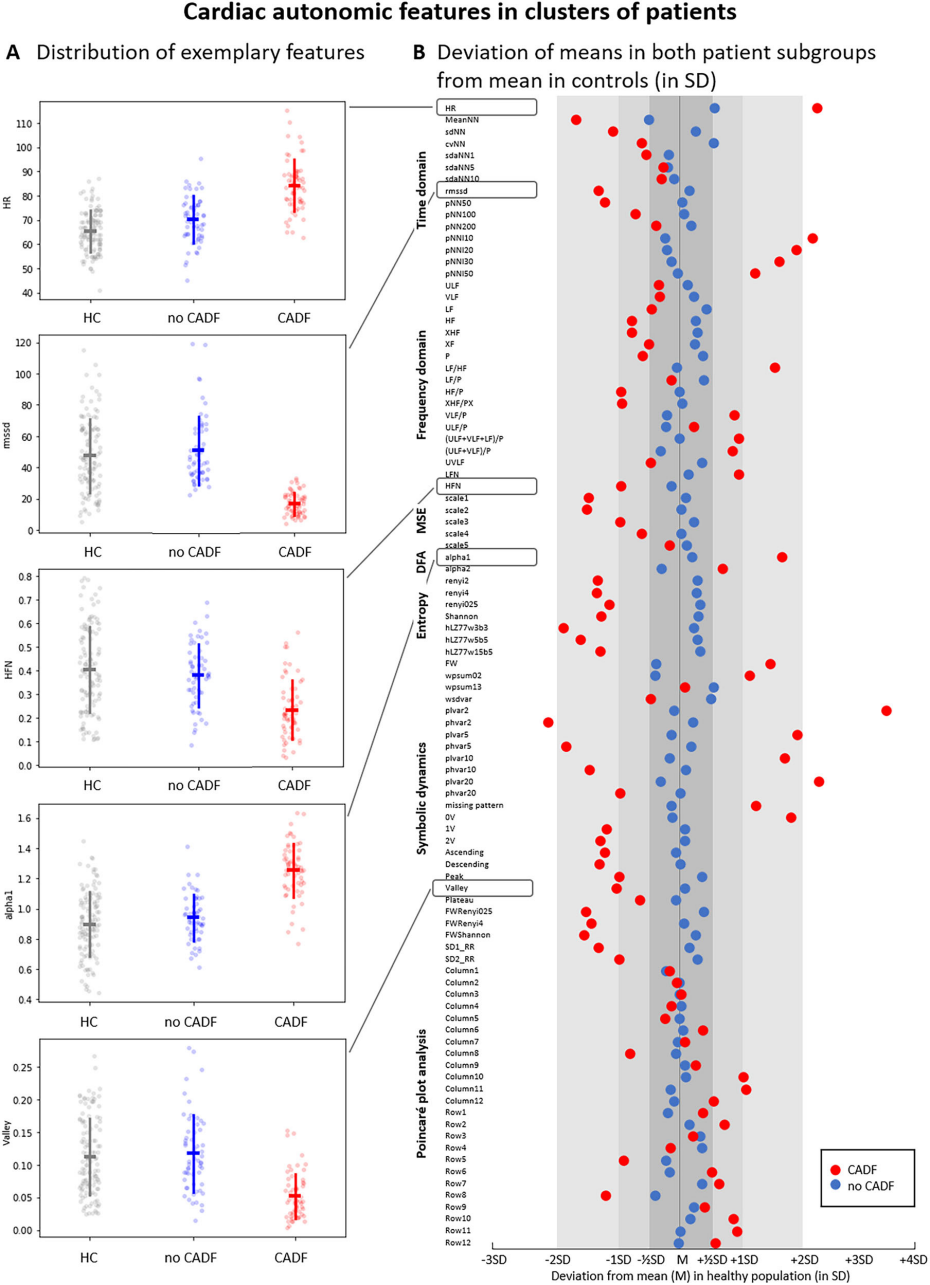
Deviation of HRV parameters in patient clusters compared to healthy controls. **A** Distribution of HRV parameters in healthy controls (HC) and patient clusters (CADF in red and no CADF in blue). Parameters shown include mean heart rate (HR), root mean square of successive interbeat intervals (RMSSD), normalized high-frequency power (HFN), short-term fluctuations in detrended fluctuation analysis (alpha1), and the proportion of the Valley symbolic pattern family. Error bars show standard deviation around the mean. **B** Deviation of the mean values of HRV parameters in the two patient clusters: red (CADF) and blue (no CADF) relative to the HC mean, expressed in standard deviation (SD) units of the control group. Abbreviations of the HRV parameters are detailed in Supplementary Table S2.

The MANOVA revealed significant differences in HRV parameters between the CADF cluster and the HC group (F = 4.524, *p* < 0.001). As shown in Fig. 3, most HRV parameters in the CADF cluster deviated several standard deviations from the HC mean (red circles, Fig. 3B), indicating CADF in these patients. In contrast, HRV parameters in the no CADF cluster remained largely within half a standard deviation of the HC mean (blue circles, Fig. 3B), with no significant group differences (F = 1.336, n.s.).

### Correlation of HRV parameters with CADF clusters

To identify the HRV parameters relevant for CADF clustering, we performed a bivariate correlation analysis between each feature and CADF group assignments (***see*** Supplementary Fig. S1). High positive correlation coefficients indicate that the higher this HRV parameter, the more likely this patient was classified in the CADF group.

The strongest positive correlations were observed in the two time-domain parameters: the percentage of consecutive NN intervals differing by less than 20 ms (pNNl20, r = 0.87) and 30 ms (pNNl30, r = 0.85), and the symbolic dynamics feature representing the number of rare word types (FW, r = 0.84). Conversely, the most pronounced negative correlations were found in two symbolic dynamics parameters: the proportion of high variability patterns (phvar5, r = −0.84) and the Renyi entropy of the word distribution (FWRenyi025, r = −0.82). In addition, the compression entropy (hLZ77w3b3) showed a significant negative correlation with the CADF clustering (r = −0.81).

### Sociodemographic and clinical differences between CADF clusters

The CADF cluster differed significantly from the no CADF cluster in age (F = 5.11, *p* = 0.001) and BMI (F = 2.24, *p* = 0.027), while no significant differences were observed in physical activity or smoking status. Patients in the CADF cluster had a significantly longer disease duration compared to those in the no CADF cluster (T = −2.86, *p* = 0.003). In terms of symptom severity, patients in the CADF cluster had higher negative and positive symptom scores as measured by SANS (F(2,116) = 2.41, *p* = 0.009) and SAPS (T = −3.31, *p* < 0.001) compared to the no CADF cluster (See Supplementary Table S3 for details).

**Fig. 4:**
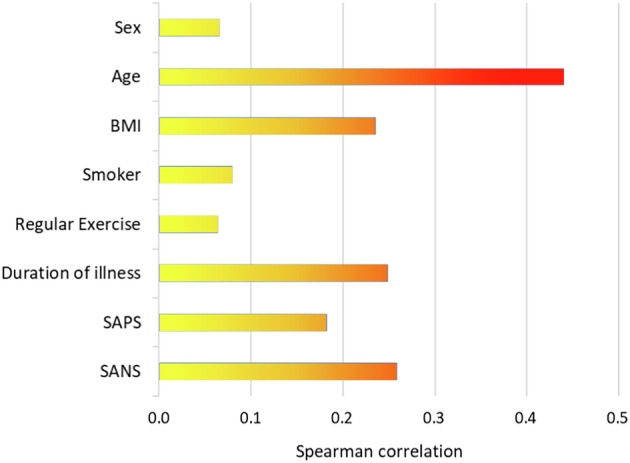
Spearman correlation coefficients of CADF cluster assignment with sociodemographic factors and psychometric scales. BMI body mass index, SAPS Scale for the Assessment of Positive Symptoms, SANS Scale for the Assessment of Negative Symptoms.

Age showed the strongest correlation with CADF classification, indicating that older patients were more likely to be identified in the CADF cluster. In addition, higher BMI, longer disease duration, and greater negative symptom severity were associated with CADF classification. Figure 4 summarizes these relationships using Spearman correlation analysis.

## Discussion

This is the first study to examine the prevalence of CADF in a large cohort of unmedicated patients with SCZ. Based on a comprehensive analysis of multiple HRV parameters, the present study used unsupervised learning to show that approximately 50% of patients have significant impairments in cardiac autonomic modulation, independent of antipsychotic medication, compared to healthy controls.

Our classification approach showed strong robustness, with clusters remaining stable across multiple statistical tests. The fact that both k-means clustering and Gaussian processes produced the same classification further validates their reliability. While k-means is optimized for well-defined, linearly separable clusters, Gaussian processes account for more complex relationships and uncertainties, confirming that the clustering patterns reflect intrinsic data structures rather than methodological artifacts.

Given the critical role of vagal and sympathetic modulation in the regulation of essential cardiac function, such as chronotropy, inotropy, dromotropy, and lusitropy, CADF results in constant stress on the cardiovascular system, placing these patients at increased risk for cardiac events, including myocardial infarction, heart failure, and sudden cardiac death^12–15^. Notably, even patients classified as “no CADF” showed considerable deviations in several HRV parameters compared to healthy controls, suggesting that the at-risk population may be even larger.

From a clinical perspective, the identification of these patients at increased cardiac autonomic risk is crucial. To ensure rigorous and unbiased identification of HRV parameters associated with CADF clustering, we used correlation-based feature analysis rather than classification-based approaches, ensuring transparency and interpretability without introducing potential classification biases.

Among the 97 HRV parameters, mean heart rate (HR) contributed significantly to clustering, which is particularly noteworthy because “simply taking the pulse” serves as an immediate indicator of cardiovascular risk^16^, as HR is an independent predictor of reduced life expectancy in both the general population and CVD patients^55,56^, with a 10 bpm increase associated with a 30% increase in mortality^57^.

However, nonlinear measures, which quantify the unpredictability and randomness of heart rate dynamics, are highly sensitive to subtle signal variations and effectively capture higher-order HRV characteristics that are commonly disrupted in SCZ^28^. Notably, both nonlinear complexity measures, particularly those derived from symbolic dynamics and entropy, as well as classical HRV parameters, such as *pNNI20*, have proven highly relevant for CADF classification. These findings highlight the importance of integrating multiple HRV metrics to enhance clustering robustness and improve risk stratification and patient identification. Thus, parameters such as *pNNl20* (time domain) and *phvar5* (symbolic dynamics) may emerge as powerful biomarkers for CADF in SCZ, warranting further validation for their role in refining cardiac risk screening in clinical practice.

The underlying biological mechanisms leading to CADF remain unclear. It is uncertain whether CADF is an intrinsic feature of SCZ or whether both conditions share common pathophysiological pathways. Interestingly, first-degree relatives of SCZ patients also exhibit reduced HRV, suggesting a possible genetic predisposition^17,58–60^. Advances in genetics have identified SCZ-associated loci that regulate cardiac function, further strengthening these links^18,19,61–64^. In addition, CADF may manifest before the clinical onset of SCZ, raising questions about its potential role as an early biomarker^65,66^.

Beyond genetic factors, several external sociodemographic and clinical variables emerge during disease progression and may influence the severity of CADF in SCZ according to individual predisposition. For instance, our data are consistent with previous findings indicating an age-related decline in cardiac autonomic function^67–69^, as the CADF cluster was significantly older compared to the no CADF subgroup. In addition, patients in the CADF cluster had higher positive and negative symptom scores, which is consistent with previous research linking symptom severity to CADF^25,70,71^. While increased positive symptom scores may reflect stressful experiences during psychotic episodes, higher negative symptom scores may be confounded by a greater proportion of chronicity in these patients^16,25^. These findings support the widely accepted view that age, disease chronicity, and symptom severity collectively contribute to CADF in SCZ^25^. Accelerated brain aging, particularly within the central autonomic network^72,73^, may underlie these associations, as it has been linked to mental disorders, particularly psychotic conditions, with longer disease duration correlating with advanced brain aging^74^.

In addition, chronic stress, lifestyle factors, and metabolic dysfunction may contribute to progressive autonomic decline over time. Thus, increased BMI was also a significant factor associated with CADF clustering, with elevated BMI levels observed in the CADF cluster. This is consistent with previous studies linking excess body fat to reduced vagal modulation in both the general population and individuals with mental disorders^75–79^. The relationship between CADF and increased BMI appears to be bidirectional: the vagus nerve plays a central role in regulating metabolic homeostasis, and autonomic dysfunction may contribute to energy imbalance^80,81^. Conversely, chronic low-grade inflammation and metabolic dysregulation associated with obesity may further exacerbate CADF, underscoring the need for targeted interventions to reduce obesity-related cardiovascular risks^79,82^.

Although smoking prevalence did not differ significantly between clusters in our cohort, its ubiquitous role as a modifiable lifestyle factor also warrants attention. Smoking is highly prevalent in patients with SCZ^83^ with well-documented effects on autonomic regulation, primarily by impairing vagal control of heart rate and increasing sympathetic activity via catecholamine release^84–87^. However, previous studies have reported conflicting results, likely due to population heterogeneity, BMI confounders, and differences in short-term versus long-term effects of smoking on autonomic function^87–91^, underscoring the need for further research.

The main findings of the present study hold several relevant implications for future research. First, cardiovascular risk profiling in mental disorders should be optimized by implementing HRV-based analyses, as current approaches focus primarily on metabolic factors^92–94^ and neglect obvious intrinsic changes in cardiac rhythmicity. While heart rate analysis has long been established as a tool for cardiac risk assessment in clinical cardiology^37,95–98^, data on prognostic relevance in mental disorders are lacking. Second, targeted interventions are needed to improve physical health and quality of life of these patients. Regular physical activity is a key approach^99–101^, but many patients with SCZ do not benefit from standard aerobic exercise in terms of cardiorespiratory fitness or HR^99,102^, highlighting the need for tailored exercise programs. Finally, patients with severe mental disorders remain less likely to receive cardioprotective treatments after cardiac events^103,104^, despite evidence that such treatments significantly reduce mortality^105^. Addressing barriers to care, such as integrating physical health needs into mental health care systems, is critical to overcoming patient nonadherence and improving access to somatic care^106,107^, ultimately improving cardiovascular outcomes in SCZ.

Several limitations of this study need to be considered. First, a major limitation of this study is its cross-sectional design. Although large-scale longitudinal studies strongly indicate that elevated HR and reduced HRV are linked to increased mortality and morbidity, this study cannot directly confirm an elevated cardiac risk in CADF patients. Moreover, we are not able to evaluate the course of CADF in our cohort. Second, our patient cohort stood out for their smoking habits. Since tobacco use affects numerous central and peripheral autonomic functions, we cannot separate the alterations due to the disease from those due to smoking^108^. Third, since no further cardiac diagnostic examinations such as echocardiography were performed, the presence of structural heart diseases could not be assessed. Further, we cannot assess a subclinical somatic disease although we have not observed any indication of an underlying inflammatory or cardiac disease during hospitalization. Fourth, HRV parameters were derived from 30-minute ECG recordings. To account for circadian influences, long-term measurements over a 24-hour period would be required. Further, despite of prior toxicological testing and precise medical records on an antipsychotic-free interval of at least 8 weeks, possible drug residues or compensatory processes by previous medication affecting autonomic function cannot be ruled out completely. Finally, a potential limitation is the possible influence of routine psychotherapeutic interventions on HRV, although no specific stress reduction therapies were administered.

In conclusion, this is the first study to investigate the extent of CADF in unmedicated patients with SCZ, showing that approximately half of the cohort had significant HRV reductions associated with increased cardiac risk. These patients were more likely to have chronic disease, greater symptom severity, older age, and higher BMI. Given the marked increase in cardiovascular mortality in SCZ, future studies should focus on refining cardiac risk prediction models and developing targeted interventions to mitigate CADF, ultimately improving long-term health outcomes in this vulnerable population.

## Supplementary information

Supplemental Material

Supplementary Figure 1

Supplementary Table 1

Supplementary Table 2

Supplementary Table 3

## Data Availability

The datasets used and/or analyzed in the current study are available from the corresponding author upon reasonable request.

## References

[1] Tiihonen, J., Lonnqvist, J., Wahlbeck, K., Klaukka, T., Niskanen, L., Tanskanen, A. & Haukka, J. 11-year follow-up of mortality in patients with schizophrenia: a population-based cohort study (FIN11 study). *Lancet (Lond., Engl.)* **374**, 620–627 (2009). 10.1016/S0140-6736(09)60742-X 19595447

[2] Bushe, C. J., Taylor, M. & Haukka, J. Mortality in schizophrenia: A measurable clinical endpoint. *J. Psychopharmacol. (Oxf., Engl.)* **24**, 17–25 (2010). 10.1177/1359786810382468 PMC2951589 20923917

[3] Laursen, T. M., Nordentoft, M. & Mortensen, P. B. Excess early mortality in schizophrenia. *Annu. Rev. Clin. Psychol.* **10**, 425–448 (2014). 24313570 10.1146/annurev-clinpsy-032813-153657

[4] Correll, C. U. et al. Prevalence, incidence and mortality from cardiovascular disease in patients with pooled and specific severe mental illness: a large-scale meta-analysis of 3,211,768 patients and 113,383,368 controls. *World Psychiatry* **16**, 163–180 (2017). 28498599 10.1002/wps.20420 PMC5428179

[5] Olfson, M., Gerhard, T., Huang, C., Crystal, S. & Stroup, T. S. Premature mortality among adults with schizophrenia in the United States. *JAMA psychiatry* **72**, 1172–1181 (2015). 26509694 10.1001/jamapsychiatry.2015.1737

[6] Ringen, P. A., Engh, J. A., Birkenaes, A. B., Dieset, I. & Andreassen, O. A. Increased mortality in schizophrenia due to cardiovascular disease - a non-systematic review of epidemiology, possible causes, and interventions. *Front. Psychiatry* **5**, 137 (2014). 25309466 10.3389/fpsyt.2014.00137 PMC4175996

[7] Gatov, E., Rosella, L., Chiu, M. & Kurdyak, P. A. Trends in standardized mortality among individuals with schizophrenia, 1993–2012: a population-based, repeated cross-sectional study. *CMAJ: Can. Med. Assoc. J. = J. de. l’Assoc. Med. Canadienne* **189**, E1177–e1187 (2017). 10.1503/cmaj.161351 PMC5602497 28923795

[8] Bauer, A. et al. Impact of myocardial salvage assessed by (99m)Tc-sestamibi scintigraphy on cardiac autonomic function in patients undergoing mechanical reperfusion therapy for acute myocardial infarction. *JACC Cardiovasc Imaging* **2**, 449–457 (2009). 19580728 10.1016/j.jcmg.2008.12.018

[9] Yperzeele, L., van Hooff, R. J., Nagels, G., De Smedt, A., De Keyser, J. & Brouns, R. Heart rate variability and baroreceptor sensitivity in acute stroke: a systematic review. *Int J. Stroke* **10**, 796–800 (2015). 26202709 10.1111/ijs.12573

[10] Vinik, A. I., Casellini, C., Parson, H. K., Colberg, S. R. & Nevoret, M. L. Cardiac autonomic neuropathy in diabetes: A predictor of cardiometabolic events. *Front Neurosci.* **12**, 591 (2018). 30210276 10.3389/fnins.2018.00591 PMC6119724

[11] Bär, K. J., Letzsch, A., Jochum, T., Wagner, G., Greiner, W. & Sauer, H. Loss of efferent vagal activity in acute schizophrenia. *J. Psychiatr. Res.* **39**, 519–527 (2005). 15992561 10.1016/j.jpsychires.2004.12.007

[12] Fukuda, K., Kanazawa, H., Aizawa, Y., Ardell, J. L. & Shivkumar, K. Cardiac innervation and sudden cardiac death. *Circ. Res.* **116**, 2005–2019 (2015). 26044253 10.1161/CIRCRESAHA.116.304679 PMC4465108

[13] Ardell, J. L. et al. Translational neurocardiology: Preclinical models and cardioneural integrative aspects. *J. Physiol.* **594**, 3877–3909 (2016). 27098459 10.1113/JP271869 PMC4945715

[14] Shivkumar, K. & Ardell, J. L. Cardiac autonomic control in health and disease. *J. Physiol.* **594**, 3851–3852 (2016). 27417670 10.1113/JP272580 PMC4945710

[15] Hadaya, J. & Ardell, JL. Autonomic Modulation for Cardiovascular Disease. *Front Physiol.* **11**, 617459 (2020). 33414727 10.3389/fphys.2020.617459 PMC7783451

[16] Bär, K. J. Cardiac autonomic dysfunction in patients with schizophrenia and their healthy relatives - A small review. *Front. Neurol.* **6**, 139 (2015). 26157417 10.3389/fneur.2015.00139 PMC4478389

[17] Bär, K.-J. et al. Autonomic dysfunction in unaffected first-degree relatives of patients suffering from schizophrenia. *Schizophr. Bull.* **36**, 1050–1058 (2010). 19366982 10.1093/schbul/sbp024 PMC2930351

[18] Trubetskoy, V. et al. Mapping genomic loci implicates genes and synaptic biology in schizophrenia. *Nature* **604**, 502–508 (2022). 35396580 10.1038/s41586-022-04434-5 PMC9392466

[19] Refisch, A. et al. A common variation in HCN1 is associated with heart rate variability in schizophrenia. *Schizophrenia Res.* **229**, 73–79 (2021). 10.1016/j.schres.2020.11.017 33221148

[20] Murgia, F. et al. Effects of smoking status, history and intensity on heart rate variability in the general population: The CHRIS study. *PLOS ONE* **14**, e0215053 (2019). 30964923 10.1371/journal.pone.0215053 PMC6456196

[21] Hassya, I., Sahroni, A., Rahayu, A. & Laksono, E. The analysis of heart rate variability properties and body mass index in representing health quality information. *Procedia Comput. Sci.* **197**, 135–142 (2022).

[22] Ballester, P. L., Romano, M. T., de Azevedo Cardoso, T., Hassel, S., Strother, S. C., Kennedy, S. H. & Frey, B. N. Brain age in mood and psychotic disorders: a systematic review and meta-analysis. *Acta Psychiatr. Scand.* **145**, 42–55 (2022). 34510423 10.1111/acps.13371

[23] Constantinides, C. et al. Brain ageing in schizophrenia: Evidence from 26 international cohorts via the ENIGMA Schizophrenia consortium. *Mol. Psychiatry* **28**, 1201–1209 (2023). 36494461 10.1038/s41380-022-01897-w PMC10005935

[24] Schnack, H. G., van Haren, N. E. M., Nieuwenhuis, M., Hulshoff Pol, H. E., Cahn, W. & Kahn, R. S. Accelerated brain aging in schizophrenia: A longitudinal pattern recognition study. *Am. J. Psychiatry* **173**, 607–616 (2016). 26917166 10.1176/appi.ajp.2015.15070922

[25] Stogios, N. et al. Autonomic nervous system dysfunction in schizophrenia: impact on cognitive and metabolic health. *NPJ Schizophr.* **7**, 22 (2021). 33903594 10.1038/s41537-021-00151-6 PMC8076312

[26] Malik, M., Hnatkova, K., Huikuri, H. V., Lombardi, F., Schmidt, G. & Zabel, M. CrossTalk proposal: Heart rate variability is a valid measure of cardiac autonomic responsiveness. *J. Physiol.* **597**, 2595–2598 (2019). 31006862 10.1113/JP277500 PMC6826215

[27] Heart rate variability: standards of measurement, physiological interpretation and clinical use. Task Force of the European Society of Cardiology and the North American Society of Pacing and Electrophysiology. *Circulation* 1996;93:1043-1065. 8598068

[28] Bär, K. J., Boettger, M. K., Koschke, M., Schulz, S., Chokka, P., Yeragani, V. K. & Voss, A. Non-linear complexity measures of heart rate variability in acute schizophrenia. *Clin. Neurophysiol.: Off. J. Int. Fed. Clin. Neurophysiol.* **118**, 2009–2015 (2007). 10.1016/j.clinph.2007.06.012 17646130

[29] Liu Y, Huang Y, Zhou J, Li G, Chen J, Xiang Z, Wu F, Wu K. Altered heart rate variability in patients with schizophrenia during an autonomic nervous test. *Front. Psychiatry* 2021;12. 10.3389/fpsyt.2021.626991 PMC8074969 33912081

[30] Costa, M., Goldberger, A. L. & Peng, C. K. Multiscale entropy analysis of complex physiologic time series. *Phys. Rev. Lett.* **89**, 068102 (2002). 12190613 10.1103/PhysRevLett.89.068102

[31] Graff, B., Graff, G., Makowiec, D., Kaczkowska, A., Wejer, D., Budrejko, S., Kozłowski, D. & Narkiewicz, K. Entropy measures in the assessment of heart rate variability in patients with cardiodepressive vasovagal syncope. *Entropy* **17**, 1007–1022 (2015).

[32] Baumert, M., Baier, V., Haueisen, J., Wessel, N., Meyerfeldt, U., Schirdewan, A. & Voss, A. Forecasting of life threatening arrhythmias using the compression entropy of heart rate. *Methods Inf. Med.* **43**, 202–206 (2004). 15136870

[33] Guzzetti, S. et al. Symbolic dynamics of heart rate variability: a probe to investigate cardiac autonomic modulation. *Circulation* **112**, 465–470 (2005). 16027252 10.1161/CIRCULATIONAHA.104.518449

[34] Babloyantz, A. & Destexhe, A. Is the normal heart a periodic oscillator? *Biol. Cybern.* **58**, 203–211 (1988). 3358954 10.1007/BF00364139

[35] Voss, A., Fischer, C., Schroeder, R., Figulla, H. R. & Goernig, M. Segmented Poincare plot analysis for risk stratification in patients with dilated cardiomyopathy. *Methods Inf. Med.* **49**, 511–515 (2010). 20526525 10.3414/ME09-02-0050

[36] Baumert, M., Voss, A. & Javorka, M. Compression based entropy estimation of heart rate variability on multiple time scales. *Annu Int Conf. IEEE Eng. Med Biol. Soc.* **2013**, 5037–5040 (2013). 24110867 10.1109/EMBC.2013.6610680

[37] Huikuri, H. V. & Stein, P. K. Heart rate variability in risk stratification of cardiac patients. *Prog. Cardiovascular Dis.* **56**, 153–159 (2013). 10.1016/j.pcad.2013.07.003 24215747

[38] Hedman, A. E. & Hartikainen, J. E. K. Has non-linear analysis of heart rate variability any practical value? *Card. Electrophysiol. Rev.* **3**, 286–289 (1999).

[39] Perkiömäki J. Heart rate variability and non-linear dynamics in risk stratification. *Front Physiol* 2011;2. 10.3389/fphys.2011.00081 PMC3210967 22084633

[40] First MB, & Gibbon, M The Structured Clinical Interview for DSM-IV Axis I Disorders (SCID-I) and the Structured Clinical Interview for DSM-IV Axis II Disorders (SCID-II). In M. J. Hilsenroth & D. L. Segal (Eds.). *Comprehensive handbook of psychological assessment* 2004;Vol. 2. Personality assessment 134–143.

[41] Andreasen, NC. The Scale for the Assessment of Negative Symptoms (SANS): conceptual and theoretical foundations. *Br. J. Psychiatry Suppl.* **155**, 49–58 (1989). 2695141

[42] Andreasen NC. Scale for the Assessment of Positive Symptoms (SAPS). 1986.

[43] Wessel, N., Voss, A., Malberg, H., Ziehmann, C., Voss, H., Schirdewan, A., Meyerfeldt, U. & Kurths, J. Nonlinear analysis of complex phenomena in cardiological data. *Herzschrittmachertherapie und Elektrophysiologie* **11**, 159–173 (2000).

[44] Eguchi, K., Aoki, R., Shimauchi, S., Yoshida, K. & Yamada, T. R-R interval outlier processing for heart rate variability analysis using wearable ECG devices. *Adv. Biomed. Eng.* **7**, 28–38 (2018). 10.1109/EMBC.2018.8513449 30441628

[45] Clifford, G. D. & Tarassenko, L. Quantifying errors in spectral estimates of HRV due to beat replacement and resampling. *IEEE Trans. Biomed. Eng.* **52**, 630–638 (2005). 15825865 10.1109/TBME.2005.844028

[46] Fortin, J. et al. Validation and verification of the task force® monitor. *Results of Clinical Studies for FDA* 01/01 2001;510.

[47] Kleiger, R. E., Stein, P. K., Bosner, M. S. & Rottman, J. N. Time domain measurements of heart rate variability. *Cardiol. Clin.* **10**, 487–498 (1992). 1504980

[48] Ori, Z., Monir, G., Weiss, J., Sayhouni, X. & Singer, D. H. Heart rate variability. Frequency domain analysis. *Cardiol. Clin.* **10**, 499–537 (1992). 1504981

[49] Penzel, T., Kantelhardt, J. W., Grote, L., Peter, J. H. & Bunde, A. Comparison of detrended fluctuation analysis and spectral analysis for heart rate variability in sleep and sleep apnea. *IEEE Trans. Biomed. Eng. Oct.* **50**, 1143–1151 (2003). 10.1109/TBME.2003.817636 14560767

[50] Ziv J, Lempel A. Universal algorithm for sequential data compression. 1977.

[51] Baumert, M., Walther, T., Baier, V., Stepan, H., Faber, R. & Voss, A. Heart rate and blood pressure interaction in normotensive and chronic hypertensive pregnancy. *Biomed. Tech. (Berl.)* **47**, 554–556 (2002). 12465234 10.1515/bmte.2002.47.s1b.554

[52] Baumert, M., Baier, V., Truebner, S., Schirdewan, A. & Voss, A. Short- and long-term joint symbolic dynamics of heart rate and blood pressure in dilated cardiomyopathy. *IEEE Trans. Biomed. Eng.* **52**, 2112–2115 (2005). 16366235 10.1109/TBME.2005.857636

[53] Lloyd, S. Least squares quantization in PCM. *IEEE Trans. Inf. Theory* **28**, 129–137 (1982).

[54] Buitinck, L. et al. API design for machinelearning software: experiences from the scikit-learn project. *European Conference on Machine Learning and Principles and Practices of Knowledge Discovery in Databases*. Prague, Czech Republic (2013).

[55] Jensen, M. T., Marott, J. L., Lange, P., Vestbo, J., Schnohr, P., Nielsen, O. W., Jensen, J. S. & Jensen, G. B. Resting heart rate is a predictor of mortality in COPD. *Eur. Respir. J.* **42**, 341–349 (2013). 23143550 10.1183/09031936.00072212

[56] Jensen, M. T., Suadicani, P., Hein, H. O. & Gyntelberg, F. Elevated resting heart rate, physical fitness and all-cause mortality: A 16-year follow-up in the Copenhagen Male Study. *Heart (Br. Card. Soc.)* **99**, 882–887 (2013). 10.1136/heartjnl-2012-303375 PMC3664385 23595657

[57] Münzel, T. et al. Heart rate, mortality, and the relation with clinical and subclinical cardiovascular diseases: results from the Gutenberg Health Study. *Clin. Res. Cardiol.* **108**, 1313–1323 (2019). 30953178 10.1007/s00392-019-01466-2 PMC6868108

[58] Berger, S., Boettger, M. K., Tancer, M., Guinjoan, S. M., Yeragani, V. K. & Bär, K. J. Reduced cardio-respiratory coupling indicates suppression of vagal activity in healthy relatives of patients with schizophrenia. *Prog. Neuropsychopharmacol. Biol. Psychiatry* **34**, 406–411 (2010). 20083149 10.1016/j.pnpbp.2010.01.009

[59] Jáuregui, O. I. et al. Autonomic nervous system activation during social cognition tasks in patients with schizophrenia and their unaffected relatives. *Cogn. Behav. Neurol.* **24**, 194–203 (2011). 22123585 10.1097/WNN.0b013e31824007e9

[60] Schulz, S., Haueisen, J., Bär, K.-J. & Andreas, V. High-resolution joint symbolic analysis to enhance classification of the cardiorespiratory system in patients with schizophrenia and their relatives. *Philos. Trans. Ser. A Math. Phys. Eng. Sci.* **373**, 20140098 (2015). 25548266 10.1098/rsta.2014.0098 PMC4281869

[61] Pardiñas, A. F. et al. Common schizophrenia alleles are enriched in mutation-intolerant genes and in regions under strong background selection. *Nat. Genet.* **50**, 381–389 (2018). 29483656 10.1038/s41588-018-0059-2 PMC5918692

[62] Ripke, S. et al. Biological insights from 108 schizophrenia-associated genetic loci. *Nature* **511**, 421–427 (2014). 25056061 10.1038/nature13595 PMC4112379

[63] Refisch, A. et al. Analysis of CACNA1C and KCNH2 risk variants on cardiac autonomic function in patients with schizophrenia. *Genes (Basel)* **13**, 2132 (2022). 36421807 10.3390/genes13112132 PMC9691174

[64] Refisch, A. et al. Associations of common genetic risk variants of the muscarinic acetylcholine receptor M2 with cardiac autonomic dysfunction in patients with schizophrenia. *World J. Biol. Psychiatry* **24**, 1–11 (2023). 35172679 10.1080/15622975.2022.2043561

[65] Latvala, A. et al. Association of Resting Heart Rate and Blood Pressure in Late Adolescence With Subsequent Mental Disorders: A Longitudinal Population Study of More Than 1 Million Men in Sweden. *JAMA Psychiatry* **73**, 1268–1275 (2016). 27784035 10.1001/jamapsychiatry.2016.2717

[66] Sandsten, K. E., Jensen, M. T., Saebye, D., Null, K., Northoff, G. & Parnas, J. Altered cardiac autonomic functioning associates with self-disorders in schizophrenia. *Schizophr. Res.* **270**, 57–62 (2024). 38865806 10.1016/j.schres.2024.06.003

[67] Abhishekh, H. A., Nisarga, P., Kisan, R., Meghana, A., Chandran, S., Trichur, R. & Sathyaprabha, T. N. Influence of age and gender on autonomic regulation of heart. *J. Clin. Monit. Comput* **27**, 259–264 (2013). 23297094 10.1007/s10877-012-9424-3

[68] Agelink, M. W., Malessa, R., Baumann, B., Majewski, T., Akila, F., Zeit, T. & Ziegler, D. Standardized tests of heart rate variability: normal ranges obtained from 309 healthy humans, and effects of age, gender, and heart rate. *Clin. Autonomic Res.* **11**, 99–108 (2001). 10.1007/BF02322053 11570610

[69] Sammito, S. & Böckelmann, I. Reference values for time- and frequency-domain heart rate variability measures. *Heart Rhythm* **13**, 1309–1316 (2016). 26883166 10.1016/j.hrthm.2016.02.006

[70] Clamor, A., Lincoln, T. M., Thayer, J. F. & Koenig, J. Resting vagal activity in schizophrenia: meta-analysis of heart rate variability as a potential endophenotype. *Br. J. Psychiatry.: J. Ment. Sci.* **208**, 9–16 (2016). 10.1192/bjp.bp.114.160762 26729841

[71] Benjamin, B. R. et al. Heart rate variability is associated with disease severity in psychosis spectrum disorders.*Progr Neuro-Psychopharmacol. Biol. Psychiatry* **111**, e014540 (2021). 10.1016/j.pnpbp.2020.110108 32946948

[72] Benarroch, E. E. The central autonomic network: Functional organization, dysfunction, and perspective. *Mayo Clin. Proc.* **68**, 988–1001 (1993). 8412366 10.1016/s0025-6196(12)62272-1

[73] Saper, C. B. The central autonomic nervous system: Conscious visceral perception and autonomic pattern generation. *Annu Rev. Neurosci.* **25**, 433–469 (2002). 12052916 10.1146/annurev.neuro.25.032502.111311

[74] Blake, K. V., Ntwatwa, Z., Kaufmann, T., Stein, D. J., Ipser, J. C. & Groenewold, N. A. Advanced brain ageing in adult psychopathology: A systematic review and meta-analysis of structural MRI studies. *J. Psychiatr. Res.* **157**, 180–191 (2023). 36473289 10.1016/j.jpsychires.2022.11.011

[75] Indumathy, J., Pal, G. K., Pal, P., Ananthanarayanan, P. H., Parija, S. C., Balachander, J. & Dutta, T. K. Association of sympathovagal imbalance with obesity indices, and abnormal metabolic biomarkers and cardiovascular parameters. *Obes. Res Clin. Pract.* **9**, 55–66 (2015). 25660176 10.1016/j.orcp.2014.01.007

[76] Laederach-Hofmann, K., Mussgay, L. & Rúddel, H. Autonomic cardiovascular regulation in obesity. *J. Endocrinol.* **164**, 59–66 (2000). 10607938 10.1677/joe.0.1640059

[77] Sztajzel, J. et al. Impact of body fat mass extent on cardiac autonomic alterations in women. *Eur. J. Clin. Invest* **39**, 649–656 (2009). 19490066 10.1111/j.1365-2362.2009.02158.x

[78] Wang, W. et al. Correlative relationship between body mass index and heart rate variability in psychiatric disorders. *Eur. Arch. Psychiatry Clin. Neurosci.*10.1007/s00406-024-01768-1 (2024). 10.1007/s00406-024-01768-1 38470538

[79] Koenig, J., Jarczok, M. N., Warth, M., Ellis, R. J., Bach, C., Hillecke, T. K. & Thayer, J. F. Body mass index is related to autonomic nervous system activity as measured by heart rate variability — A replication using short term measurements. *J. Nutr. Health Aging***18**, 300–302 (2014).24626758 10.1007/s12603-014-0022-6

[80] Borgmann, D. & Fenselau, H. Vagal pathways for systemic regulation of glucose metabolism. *Semin. Cell Dev. Biol.* **156**, 244–252 (2024). 37500301 10.1016/j.semcdb.2023.07.010

[81] Teckentrup, V., Neubert, S., Santiago, J. C. P., Hallschmid, M., Walter, M. & Kroemer, N. B. Non-invasive stimulation of vagal afferents reduces gastric frequency. *Brain Stimul.* **13**, 470–473 (2020). 31884186 10.1016/j.brs.2019.12.018

[82] Soares, F. H. R., Furstenberger, A. B., Carvalho, L. C. S., Melo, M. Y. S., Lima, J. G. & de Sousa, M. B. C. Can body mass index identify cardiac autonomic dysfunction in women who are apparently healthy? *Women Health* **60**, 168–178 (2020). 31096889 10.1080/03630242.2019.1613472

[83] Ding, J. B. & Hu, K. Cigarette smoking and schizophrenia: Etiology, clinical, pharmacological, and treatment implications. *Schizophr. Res Treat.* **2021**, 7698030 (2021). 10.1155/2021/7698030 PMC8687814 34938579

[84] Tayade, M. C. & Kulkarni, N. B. The effect of smoking on the cardiovascular autonomic functions: a cross sectional study. *J. Clin. Diagn. Res* **7**, 1307–1310 (2013). 23998052 10.7860/JCDR/2013/5526.3133 PMC3749622

[85] Hayano, J., Yamada, M., Sakakibara, Y., Fujinami, T., Yokoyama, K., Watanabe, Y. & Takata, K. Short- and long-term effects of cigarette smoking on heart rate variability. *Am. J. Cardiol.* **65**, 84–88 (1990). 2294686 10.1016/0002-9149(90)90030-5

[86] Rajendra Acharya, U., Paul Joseph, K., Kannathal, N., Lim, C. M. & Suri, J. S. Heart rate variability: a review. *Med Biol. Eng. Comput* **44**, 1031–1051 (2006). 17111118 10.1007/s11517-006-0119-0

[87] Niedermaier, O. N., Smith, M. L., Beightol, L. A., Zukowska-Grojec, Z., Goldstein, D. S. & Eckberg, D. L. Influence of cigarette smoking on human autonomic function. *Circulation* **88**, 562–571 (1993). 8339419 10.1161/01.cir.88.2.562

[88] Makhoul, N. et al. Effects of cigarette smoking on cardiac autonomic responses: A cross-sectional study. *Int. J. Environ. Res. Public Health* **17**, 8571 (2020). 33227904 10.3390/ijerph17228571 PMC7699137

[89] Dinas, P. C., Koutedakis, Y. & Flouris, A. D. Effects of active and passive tobacco cigarette smoking on heart rate variability. *Int. J. Cardiol.* **163**, 109–115 (2013). 22100604 10.1016/j.ijcard.2011.10.140

[90] Murata, K., Landrigan, P. J. & Araki, S. Effects of age, heart rate, gender, tobacco and alcohol ingestion on R-R interval variability in human ECG. *J. Auton. Nerv. Syst.* **37**, 199–206 (1992). 1587997 10.1016/0165-1838(92)90041-e

[91] Kageyama, T., Nishikido, N., Honda, Y., Kurokawa, Y., Imai, H., Kobayashi, T., Kaneko, T. & Kabuto, M. Effects of obesity, current smoking status, and alcohol consumption on heart rate variability in male white-collar workers. *Int Arch. Occup. Environ. Health* **69**, 447–454 (1997). 9215932 10.1007/s004200050173

[92] Correll, C. U. et al. Cardiometabolic risk in patients with first-episode schizophrenia spectrum disorders: baseline results from the RAISE-ETP study. *JAMA psychiatry* **71**, 1350–1363 (2014). 25321337 10.1001/jamapsychiatry.2014.1314

[93] Vancampfort, D., Wampers, M., Mitchell, A. J., Correll, C. U., De Herdt, A., Probst, M. & De Hert, M. A meta-analysis of cardio-metabolic abnormalities in drug naïve, first-episode and multi-episode patients with schizophrenia versus general population controls. *World Psychiatry.* **12**, 240–250 (2013). 24096790 10.1002/wps.20069 PMC3799255

[94] Aoki, R., Saito, T., Ninomiya, K., Shimasaki, A., Ashizawa, T., Ito, K., Ikeda, M. & Iwata, N. Shared genetic components between metabolic syndrome and schizophrenia: Genetic correlation using multipopulation data sets. *Psychiatry Clin. Neurosci.* **76**, 361–366 (2022). 35536160 10.1111/pcn.13372 PMC9546074

[95] Goldenberg, I. et al. Heart rate variability for risk assessment of myocardial ischemia in patients without known coronary artery disease: the HRV-DETECT (heart rate variability for the detection of myocardial ischemia) study. *J Am. Heart. Assoc* **8**, e014540 (2019). 31838969 10.1161/JAHA.119.014540 PMC6951049

[96] Heldeweg, M. L. A., Liu, N., Koh, Z. X., Fook-Chong, S., Lye, W. K., Harms, M. & Ong, M. E. H. A novel cardiovascular risk stratification model incorporating ECG and heart rate variability for patients presenting to the emergency department with chest pain. *Crit. Care* **20**, 179 (2016). 27286655 10.1186/s13054-016-1367-5 PMC4903012

[97] Sessa, F. et al. Heart rate variability as predictive factor for sudden cardiac death. *Aging* **10**, 166–177 (2018). 29476045 10.18632/aging.101386 PMC5842851

[98] Shaffer, F. & Ginsberg, J. P. An overview of heart rate variability metrics and norms. *Front Public Health* **5**, 258–258 (2017). 29034226 10.3389/fpubh.2017.00258 PMC5624990

[99] Herbsleb, M. et al. The influence of continuous exercising on chronotropic incompetence in multi-episode schizophrenia. *Front. Psychiatry* **10**, 90 (2019). 30918486 10.3389/fpsyt.2019.00090 PMC6424878

[100] Routledge, F. S., Campbell, T. S., McFetridge-Durdle, J. A. & Bacon, S. L. Improvements in heart rate variability with exercise therapy. *Can. J. Cardiol.* **26**, 303–312 (2010). 20548976 10.1016/s0828-282x(10)70395-0 PMC2903986

[101] Ostermann, S. et al. Exercise reveals the interrelation of physical fitness, inflammatory response, psychopathology, and autonomic function in patients with schizophrenia. *Schizophr. Bull.* **39**, 1139–1149 (2012). 22966149 10.1093/schbul/sbs085 PMC3756770

[102] Herbsleb, M., Mühlhaus, T. & Bär, K. J. Differential cardiac effects of aerobic interval training versus moderate continuous training in a patient with schizophrenia: a case report. *Front. Psychiatry* **5**, 119 (2014). 25221528 10.3389/fpsyt.2014.00119 PMC4148625

[103] Schulman-Marcus, J. et al. Comparison of trends in incidence, revascularization, and in-hospital mortality in ST-elevation myocardial infarction in patients with versus without severe mental illness. *Am. J. Cardiol.* **117**, 1405–1410 (2016). 26956637 10.1016/j.amjcard.2016.02.006

[104] Jakobsen, L. et al. Severe mental illness and clinical outcome after primary percutaneous coronary intervention. *Am. J. Cardiol.* **120**, 550–555 (2017). 28645474 10.1016/j.amjcard.2017.05.021

[105] Kugathasan, P., Horsdal, H. T., Aagaard, J., Jensen, S. E., Laursen, T. M. & Nielsen, R. E. Association of secondary preventive cardiovascular treatment after myocardial infarction with mortality among patients with schizophrenia. *JAMA Psychiatry* **75**, 1234–1240 (2018). 30422158 10.1001/jamapsychiatry.2018.2742 PMC6583028

[106] Woodhead, C. et al. Cardiovascular disease treatment among patients with severe mental illness: A data linkage study between primary and secondary care. *Br. J. Gen. Pract.* **66**, e374–e381 (2016). 27114210 10.3399/bjgp16X685189 PMC4871302

[107] Mitchell, A. J., Vancampfort, D., Sweers, K., van Winkel, R., Yu, W. & De Hert, M. Prevalence of metabolic syndrome and metabolic abnormalities in schizophrenia and related disorders–a systematic review and meta-analysis. *Schizophr. Bull.* **39**, 306–318 (2013). 22207632 10.1093/schbul/sbr148 PMC3576174

[108] Omerbegovic, M. Linear short-term heart rate variability parameters of subjects tobacco cigarette smokers and subjects nonsmokers in preoperative period. *Med Arch.* **71**, 12–15 (2017). 28428666 10.5455/medarh.2017.71.12-15 PMC5364793

