# Supplementary material for: Characterization of cardiac autonomic dysfunction in acute Schizophrenia: a cluster analysis of heart rate variability parameters
Source: Schizophrenia (Heidelb). 2025 Mar 8;11(1):40. doi: 10.1038/s41537-025-00589-y (PMC11890564; doi:10.1038/s41537-025-00589-y)
Supplement: Supplementary file 1 — Supplemental Material [file 41537_2025_589_MOESM1_ESM.docx]

**Cardiac autonomic phenotypes in acute schizophrenia: A cluster analysis in 119 unmedicated patients**

Alexander Refisch, Andy Schumann, Yubraj Gupta, Steffen Schulz, Andreas Voss, Berend Malchow, Karl-Jürgen Bär

**Supplemental Materials**

***Supplementary Methods:*** Analytical domains for assessing heart rate dynamics (see below).

***Supplementary Table S1:*** Sociodemographic data of healthy controls and patients with schizophrenia

***Supplementary Table S2:*** Overview of features characterizing heart rate dynamics

***Supplementary Table S3:*** Differences in features characterizing heart rate dynamics between healthy controls and the CADF clusters

***Supplementary Table S4:*** Differences in sociodemographic and clinical data between patients with and without CADF

***Supplementary Figure S1****:* Contribution of features to clustering results. Bars indicate Spearman correlation coefficients between each cardiac autonomic index and the cluster assignment, distinguishing between groups the CADF cluster (No CADF=0/CADF=1). Strong positive Spearman rhos indicate that elevated values of a particular index tend to lead to assignment into the CADF cluster.

**Supplementary Methods: Analytical domains for assessing heart rate dynamics**

**Time domain analysis of heart rate variability (HRV):** Statistical measurement of the intervals between consecutive heartbeats, referred to as NN intervals (normal-to-normal intervals) or RR intervals (the interval between two consecutive R waves of the QRS signal on an electrocardiogram). This method provides insight into the autonomic regulation of the heart by the sympathetic and parasympathetic nervous systems.

**Frequency domain of HRV:** This analysis is based on the concept that additional information can be obtained by dividing the HRV signal into different frequency bands and then evaluating them using spectral analysis ^1^.

Mathematical frequency analysis techniques, such as Fast Fourier Transformation, decompose the recorded time signal, such as the ECG, into its individual oscillatory components (fundamental frequencies). This process converts time-based NN intervals into frequency-based data, allowing the relative proportions of different fundamental frequencies within the overall spectrum to be determined.

The high frequency (HF) parameter, indicative of vagal activity, ranges from 0.15 to 0.4 Hertz (Hz) and represents respiratory sinus arrhythmia ^2, 3^. After vagal stimulation, a cardiac response occurs within a maximum of 400 ms, peaking 1 to 2 heartbeats later. The low frequency (LF) parameter, ranging from 0.04 to 0.15 Hz, includes both sympathetic and vagal components and also reflects baroreflex oscillations ^4^. The ratio of low-frequency sympathetic to high-frequency vagal impulses, expressed as the LF/HF parameter, is incorporated into our scoring system.

**Mulitiscale analysis:** A major advantage of multiscale analysis is that it evaluates complexity not as the predictability of a time series, but as "meaningful structural diversity" ^5^. In addition, multiscale analysis of entropy (MSE) reveals the dependence of entropy measurements on the respective time scale.

To apply multiscale analysis, the original time series must first be transformed by a coarse-graining process. A stochastic process is considered at less fine time intervals to smooth the parameters and improve comparability. Successive data points are averaged in non-overlapping windows of increasing length (τ) to transform the original sequence of N data points into coarse-grained time series. For each time series [x1,...,xi,...,xN] and scale factor (τ), each element of the coarse-grained time series Y is calculated as follows:

Y(j)=τ1​∑i=(j−1)τ+1jτ​x(i)

The length of each coarse-grained time series is N/τ. When the scale factor is 1, the coarse-grained time series is equal to the original time series.

The Sample Entropy (SampEn) algorithm developed by Richman and Moorman ^6^ was used to analyze the entropy. To compute SampEn, for each time series y, the distance between the vectors x1 and xj in the phase space x(1), x(2), ..., x(N - m + 1) is determined, where m ≤ N is a positive integer representing the dimension of the time series ^7^. All distances within a given radius r are measured and normalized to N - m + 1. This is done twice, for m and m+1 The segment length m and the tolerance level r must be specified before the SampEn algorithm is applied ^8^. SampEn is then calculated for each coarse-grained time series and plotted as a function of the corresponding scale value.

SampEn identifies repeating patterns within the time series and provides insight into the regularity and complexity of the data set. Higher SampEn values indicate less regularity, reflecting greater complexity.

**Detrended fluctuation analysis:** Detrended fluctuation analysis (DFA) is a widely used method for quantifying correlations within non-stationary time series ^9^. In this method, the time series is analyzed for random, repeating segments. To compute DFA, individual RR interval time series (with N total RR intervals) are first integrated by subtracting the average RR interval (RRIavr) from each RR interval (RRI(i)), where i ranges from 1 to N. The average RR interval is subtracted from the average RR interval (RRIavr).

The integrated time series y(i) is then divided into equal, non-overlapping segments of length n ^10^. A least squares regression line is then fitted to each segment, representing the local trend yn(i) within that segment. The local trend is removed by subtracting yn(i) from each segment y(i), resulting in detrended time series segments of length n. For each segment length n, the fluctuation function F(n) is calculated as follows

F(n)=1N∑i=1N[y(i)-yn(i)]2F(n)=N1∑i=1N[y(i)-yn(i)]2

As the segment length n increases, F(n) typically increases as well ^11^. Finally, the self-similarity parameter or scaling exponent α is determined, which is defined as the slope of the log-log plot of F(n) versus n. Both F(n) and n are logarithmized, so α corresponds to the slope of the linear fit in the log-log plot ^9^.

The parameter alpha1 calculates short-term fractal scaling properties over the range of 4 to 16 RR intervals ^11^. An optimal value for α is 1.0, indicating that HRV signals are 50% random and 50% structured, reflecting both an underlying stability in the regulatory system and a rapid responsiveness to change. A value greater than 1 indicates greater stability and potential compensatory processes, while a value less than 1 indicates increased randomness, implying less coordinated interactions of the regulatory system.

**Compression entropy:** Another nonlinear analysis method is compression entropy, which is calculated from the ratio of the length of an original time series to its compressed version. The entropy, or complexity, of a given text or data set is defined as the smallest algorithm capable of generating that text or data set.

For lossless data compression, this study used the LZ77 algorithm introduced by Lempel and Ziv in 1977, which uses a sliding window technique ^12^. Assuming that the time series to be compressed is very large (L→∞), the compression entropy index (Hc) is calculated as the ratio of the compressed length (M) to the original time series length (L): Hc = M/L.

Thus, heart rate compression entropy (Hc) quantifies the extent to which a time series of RR intervals can be compressed by detecting repetitive sequences. A higher Hc index indicates a more complex data set.

**Symbolic Dynamics:** Classical time and frequency domain analysis methods are often insufficient to describe the complex fluctuations and nonlinear behavior of heart rate. Nonlinear dynamics methods derived from chaos theory can provide additional insight into the state and temporal evolution of the system. In particular, symbolic dynamics allows the analysis of the temporal dynamics of HRV by comparing each heartbeat with the previous one. The RR tachogram is transformed into a sequence of symbols based on the changes in the RR intervals ^13^.

The following rules have been used to transform time series into symbols ^14^:

Symbol "0": μ < RRi ≤ (1 + a) * μ

Symbol "1": (1 + a) * μ < RRi < ∞

Symbol "2": (1 - a) * μ < RRi ≤ μ

Symbol "3": 0 < RRi ≤ (1 - a) * μ

Where μ is the mean of all RR intervals in the series, a is a threshold (a=0.1), RRi is the RR interval at time i, and N is the number of RR intervals.

Based on these rules, each RR interval is assigned a symbol from the alphabet A = [0, 1, 2, 3]. A word sequence is then derived from the symbol sequence, with each word consisting of three consecutive symbols, resulting in 64 possible word types. The frequency of each word type is plotted in a histogram. Although this transformation loses some detailed information, it allows quantifying the dynamics of the time series ^15^. The distribution of word types reveals the nonlinear properties and complexity of the system.

The frequency of word types is evaluated using parameters such as Rényi entropy, which measures the entropy of the word distribution in the histogram and provides a measure of the complexity and predictability of the system.

Voss et al. proposed another approach to analyze high and low variability ^16^. This method uses a reduced alphabet with only the symbols "0" and "1". The symbol "0" indicates a difference between two consecutive RR intervals within a defined period (e.g., 5, 10, 20, 50, 100 ms), while "1" indicates a difference outside this period ^17^. Only words consisting of six identical symbols are considered, with "111111" indicating high variability and "000000" indicating low variability. The parameter Plvar, based on this approach, includes the respective time limit, e.g. Plvar10 for a 10 ms limit.

Another method distributes the symbols over 6 levels from 0 to 5 ^18^, forming words of 3 symbols. The parameter "ascending" indicates the proportion of words with three consecutive ascending symbols, while "descending" indicates the proportion of words with three consecutive descending symbols ^10^.

**Poincaré plot analysis:** The Poincaré plot ^19^ is a prominent method of nonlinear HRV analysis. By plotting each RR interval against the next RR interval, this technique visually represents the correlation between successive intervals, creating a two-dimensional scatter plot. This plot typically forms an oval shape influenced by sympathetic and vagal activity, providing detailed insight into dynamic cardiac changes.

Qualitative assessment of the plot shape allows for quick and accurate detection of artifacts and outliers. For quantitative analysis, an ellipse centered on the mean of the RR intervals is superimposed on the plot ^20^. The standard deviations of the perpendicular distances of the RRi/RRi+1 points to the short and long axes of the ellipse define the parameters SD1 and SD2, respectively. SD1 is more sensitive to rapid, high-frequency changes in heart rate, while SD2 quantifies long-term HRV.

In the segmented Poincaré plot analysis method introduced by Voss et al., a linear regression line is added to the scatter plot ^20^. The point cloud is then rotated around the center by an angle α, which is derived from the slope of the regression line relative to the x-axis. A 12x12 grid of equal rectangles is superimposed on the rotated plot, with SD1 and SD2 representing the height and width of each rectangle, respectively. The number of points within each rectangle is analyzed relative to the total number of points, determining the probability of occurrence for each column and row ^21^.

**References**

**1.** Heart rate variability: standards of measurement, physiological interpretation and clinical use. Task Force of the European Society of Cardiology and the North American Society of Pacing and Electrophysiology. *Circulation* Mar 1 1996;93(5):1043-1065.

**2.** Rechlin T. [The significance of heart rate analysis in psychiatric questions]. *Fortschr Neurol Psychiatr* Mar 1995;63(3):106-120.

**3.** Agelink MW, Malessa R, Baumann B, Majewski T, Akila F, Zeit T, Ziegler D. Standardized tests of heart rate variability: normal ranges obtained from 309 healthy humans, and effects of age, gender, and heart rate. *Clin Auton Res* Apr 2001;11(2):99-108.

**4.** de Boer RW, Karemaker JM, Strackee J. Relationships between short-term blood-pressure fluctuations and heart-rate variability in resting subjects. II: A simple model. *Med Biol Eng Comput* Jul 1985;23(4):359-364.

**5.** Costa M, Goldberger AL, Peng CK. Multiscale entropy analysis of complex physiologic time series. *Physical review letters* Aug 5 2002;89(6):068102.

**6.** Richman JS, Moorman JR. Physiological time-series analysis using approximate entropy and sample entropy. *Am J Physiol Heart Circ Physiol* Jun 2000;278(6):H2039-2049.

**7.** Valenza G, Nardelli M, Bertschy G, Lanatà A, Barbieri R, Scilingo EP. Maximal-radius multiscale entropy of cardiovascular variability: a promising biomarker of pathological mood states in bipolar disorders. *Annu Int Conf IEEE Eng Med Biol Soc* 2014;2014:6663-6666.

**8.** Schulz S, Koschke M, Bär KJ, Voss A. The altered complexity of cardiovascular regulation in depressed patients. *Physiological measurement* Mar 2010;31(3):303-321.

**9.** Penzel T, Kantelhardt JW, Grote L, Peter JH, Bunde A. Comparison of detrended fluctuation analysis and spectral analysis for heart rate variability in sleep and sleep apnea. *IEEE Trans Biomed Eng* Oct 2003;50(10):1143-1151.

**10.** Schulz S, Koschke M, Bar KJ, Voss A. The altered complexity of cardiovascular regulation in depressed patients. *Physiological measurement* Mar 2010;31(3):303-321.

**11.** Peng CK, Havlin S, Stanley HE, Goldberger AL. Quantification of scaling exponents and crossover phenomena in nonstationary heartbeat time series. *Chaos (Woodbury, NY)* 1995;5(1):82-87.

**12.** Ziv J, Lempel A. A universal algorithm for sequential data compression. *IEEE Transactions on Information Theory* 1977;23(3):337-343.

**13.** Baumert M, Walther T, Baier V, Stepan H, Faber R, Voss A. [Heart rate and blood pressure interaction in normotensive and chronic hypertensive pregnancy]. *Biomed Tech (Berl)* 2002;47 Suppl 1 Pt 2:554-556.

**14.** Voss A, Kurths J, Kleiner HJ, et al. The application of methods of non-linear dynamics for the improved and predictive recognition of patients threatened by sudden cardiac death. *Cardiovascular research* Mar 1996;31(3):419-433.

**15.** Kurths J, Voss A, Saparin P, Witt A, Kleiner HJ, Wessel N. Quantitative analysis of heart rate variability. *Chaos (Woodbury, NY)* Mar 1995;5(1):88-94.

**16.** Bauernschmitt R, Malberg H, Wessel N, Kopp B, Schirmbeck EU, Lange R. Impairment of cardiovascular autonomic control in patients early after cardiac surgery. *Eur J Cardiothorac Surg* Mar 2004;25(3):320-326.

**17.** Voss A, Wessel N, Baier V, Osterziel K, Kurths J, Dietz R. Symbolic Dynamics - a Powerful Tool in Non-Invasive Biomedical Signal Processing. 08/13 2000.

**18.** Porta A, Guzzetti S, Montano N, Furlan R, Pagani M, Malliani A, Cerutti S. Entropy, entropy rate, and pattern classification as tools to typify complexity in short heart period variability series. *IEEE Trans Biomed Eng* Nov 2001;48(11):1282-1291.

**19.** Babloyantz A, Destexhe A. Is the normal heart a periodic oscillator? *Biological cybernetics* 1988;58(3):203-211.

**20.** Voss A, Fischer C, Schroeder R, Figulla HR, Goernig M. Segmented Poincare plot analysis for risk stratification in patients with dilated cardiomyopathy. *Methods of information in medicine* 2010;49(5):511-515.

**21.** Seeck A, Rademacher W, Fischer C, Haueisen J, Surber R, Voss A. Prediction of atrial fibrillation recurrence after cardioversion-interaction analysis of cardiac autonomic regulation. *Med Eng Phys* Mar 2013;35(3):376-382.
