# Supplementary figures and images for: Characterization of cardiac autonomic dysfunction in acute Schizophrenia: a cluster analysis of heart rate variability parameters
Source: Schizophrenia (Heidelb). 2025 Mar 8;11(1):40. doi: 10.1038/s41537-025-00589-y (PMC11890564; doi:10.1038/s41537-025-00589-y)

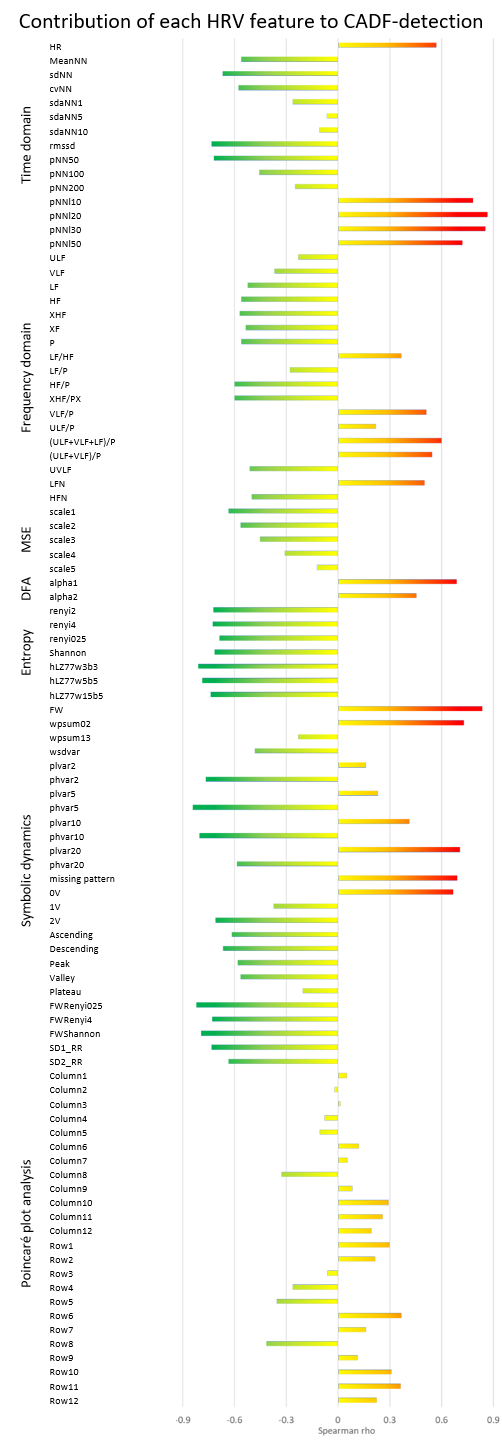

Supplement: Supplementary file 2 — Supplementary Figure 1 [file 41537_2025_589_MOESM2_ESM.tif]
